# Psychological capital and pre-competition anxiety among adolescent basketball players: the chain mediating roles of mental fatigue and achievement motivation

**DOI:** 10.3389/fpsyg.2026.1800210

**Published:** 2026-03-06

**Authors:** Xuefeng Liu, Qian Ge, Heng Liu

**Affiliations:** 1 Physical Rehabilitation Center, Sichuan Sports College, Chengdu, China; 2 College of Physical Education, Chongqing University, Chongqing, China

**Keywords:** achievement motivation, adolescent basketball players, chain mediation, mental fatigue, pre-competition anxiety, psychological capital

## Abstract

**Objective:**

This study aimed to explore the association mechanism between psychological capital and pre-competition anxiety in adolescent basketball players, and to verify the independent and chain mediating roles of mental fatigue and achievement motivation.

**Methods:**

A total of 510 Chinese adolescent basketball players from Sichuan Province completed the Psychological Capital Questionnaire, Athlete Burnout Questionnaire, Individual Differences in Achievement Tendency Scale, and Competitive State Anxiety Inventory-2 24 h pre-competition.

**Results:**

Psychological capital was positively correlated with state self-confidence and negatively correlated with cognitive/somatic anxiety and mental fatigue (*p* < 0.01); mental fatigue was positively associated with state anxiety (*p* < 0.01); achievement motivation correlated positively with state self-confidence and negatively with state anxiety (*p* < 0.05). Mental fatigue (indirect effect = −0.096) and achievement motivation (indirect effect = −0.039) independently partially mediated the psychological capital-pre-competition anxiety link; achievement motivation also mediated psychological capital and state self-confidence (indirect effect = 0.078). Additionally, mental fatigue and achievement motivation exerted a chain mediating effect (indirect effect = 0.011) between psychological capital and pre-competition anxiety.

**Conclusion:**

Psychological capital is directly negatively associated with pre-competition anxiety in adolescent basketball players and indirectly alleviates anxiety by reducing mental fatigue and optimizing achievement motivation. Based on the above findings, targeted psychological interventions for different subgroups of adolescent basketball players are proposed as follows: improve psychological capital and reduce failure-avoidance motivation for younger athletes; address reduced sense of accomplishment for centers; strengthen pre-competition physical relaxation training for females; and provide more competitive opportunities for substitutes to build self-confidence.

## Introduction

As a high-intensity, highly confrontational collective sport, basketball places extremely high demands on athletes’ technical and tactical skills as well as psychological qualities (de Souza et al., 2024). In recent years, the training system for adolescent basketball reserve talents in China has been gradually improved, but the problem of lagging development of athletes’ psychological abilities has become increasingly prominent (Yu and Yang, 2025). In international adolescent competitions, Chinese players are not inferior to their opponents in technical ability, but they often fail to perform due to excessive pre-competition anxiety, which has become a key factor restricting the improvement of competitive level.

Psychological capital (Goel and Wani, 2024), as a core element of individuals’ positive psychological state, includes four dimensions: self-efficacy, resilience, hope, and optimism, and plays an important role in alleviating athletes’ anxiety. However, long-term high-intensity training is likely to induce athletes’ mental fatigue (Santos et al., 2025), which is manifested as emotional exhaustion, reduced sense of accomplishment, and negative evaluation of sports, and further exacerbates pre-competition anxiety (Martínez-Gallego et al., 2022). Achievement motivation, as the internal motivation driving athletes to pursue success, has structural characteristics that directly affect the level of pre-competition anxiety (Junli et al., 2021). However, existing studies mostly focus on examining the direct effect of psychological capital on pre-competition anxiety (Anwari et al., 2025; Zhang et al., 2024), or exploring the independent role of a single mediating variable (such as mental fatigue or achievement motivation) (Oliver et al., 2025; Kenioua and El-Kadder, 2016), making it difficult to reveal the interaction mechanism between various psychological variables.

As a positive personal resource, the role of psychological capital may not be single or parallel (Kuhlmann and Süß, 2024). Psychological capital may protect athletes’ psychological functions by reducing mental exhaustion caused by long-term training (Yu et al., 2025); the protected psychological functions, in turn, help maintain or stimulate their healthy achievement motivation oriented towards pursuing success (Ahmad, 2021). Ultimately, this healthy motivational state is the psychological foundation for resisting athletes’ pre-competition anxiety (Sánchez et al., 2023). Existing studies have shown that psychological capital can protect individuals’ motivational functions by alleviating mental fatigue (Tian et al., 2020), while the alleviation of mental fatigue can further strengthen the success-pursuing tendency in achievement motivation (Milyavskaya et al., 2021), and ultimately, the optimization of achievement motivation can reduce athletes’ pre-competition anxiety (Yang et al., 2020). Building on the conservation of resources theory and self-determination theory (Ahmad, 2021; Milyavskaya et al., 2021), psychological capital—as a positive psychological resource—can reduce athletes’ mental fatigue by safeguarding their psychological resources, thereby satisfying their basic psychological needs (i.e., competence, autonomy, and relatedness) as proposed by Self-Determination Theory. Since the satisfaction of these needs is the core premise for developing healthy achievement motivation oriented toward success, we propose a sequential chain where psychological capital influences mental fatigue, which in turn shapes achievement motivation, ultimately impacting pre-competition anxiety. This provides indirect evidence for the path of “psychological capital → mental fatigue → achievement motivation → pre-competition anxiety” in athletes, but it has not been verified in the group of adolescent basketball players. Recent studies have confirmed that motivation, mental skills, physical activity, and sleep quality are closely related to the psychological function of adolescent athletes (El Oirdi et al., 2023a; El Oirdi et al., 2024), and achievement motivation, as the core internal motivation, is an important link between psychological resources and competitive anxiety (Junli et al., 2021).

Therefore, taking Chinese adolescent basketball players in Sichuan Province as the research object, this study aims to construct and verify a chain mediation model with mental fatigue and achievement motivation as dual mediators, clarify the direct and indirect effects of psychological capital on pre-competition anxiety, and reveal the internal influence mechanism of psychological capital on pre-competition anxiety, so as to make up for the deficiency of single mediator research in existing studies. It provides a theoretical basis for formulating sequential psychological intervention programs from the perspective of “resource-loss-motivation.” Taking adolescent basketball players as the research object, the following hypotheses are proposed.

*H1*: There are significant pairwise correlations among the four variables of psychological capital, mental fatigue, achievement motivation, and pre-competition anxiety.

*H2*: Mental fatigue and achievement motivation, respectively, play independent mediating roles between psychological capital and pre-competition anxiety (psychological capital can alleviate pre-competition anxiety both by reducing mental fatigue and optimizing the structure of achievement motivation).

*H3*: Mental fatigue and achievement motivation play a chain mediating role between psychological capital and pre-competition anxiety.

## Materials and methods

### Participants

This study was approved by the Human Experiment Ethics Committee of Sichuan Sports College (approval number: SCSC202409). Adolescent basketball players from 21 cities and prefectures in Sichuan Province, China, were selected as the research subjects.

*Inclusion criteria*: Aged 13–18 years with training experience of ≥2 years; participated in the 2024 Sichuan Provincial Adolescent Basketball Championship on behalf of their respective cities/prefectures; free from major physical or mental illnesses. The study was conducted in accordance with the Declaration of Helsinki, and written informed consent was obtained from both the participants and their guardians. Exclusion criteria: Unable to participate in the competition due to injury or illness during the study period; incomplete questionnaire responses (missing ≥3 items) or obvious regular response patterns (selecting the same option for 10 consecutive questions).

*A priori* power analysis was conducted using G*Power 3.1 to determine the required sample size for testing the chain mediation model. For a multiple regression model with 4 predictors (psychological capital, mental fatigue, achievement motivation, and covariates), an effect size of *f*^2^ = 0.15 (medium effect), *α* = 0.05, and power = 0.95, the required sample size was 320. *Post hoc* power analysis confirmed that the final sample size (*n* = 510) provided sufficient power (1−*β* = 0.99) to detect small indirect effects (*f*^2^ = 0.02) in the SEM. A total of 520 athletes were initially enrolled, and 510 valid questionnaires were recovered, with an effective recovery rate of 98.1%—exceeding the minimum sample size required for the study. Based on the demographic characteristics (gender, age) and sports-related attributes (playing position, match status, training years) of the adolescent basketball players, and with reference to the grouping standards for Chinese adolescent basketball competitions and the actual sports situation of the athletes, the subjects were divided into subgroups by gender, age group (U13–U15/U16–U18), playing position (guard/forward/center/swingman), match status (starter/substitute), and training years (2–3 years/4–5 years/≥6 years). Descriptive statistics and inter-group difference tests were conducted after grouping (Table 1).

**Table 1:** Basic information of participants.

Demographic characteristic	Group	*n*	%	Key difference results
Gender	Male	435	85.3	Somatic state anxiety: *p* < 0.05 (female > male)
	Female	75	14.7	
Age group	U13-U15 (younger age group)	312	61.2	Older age group > younger age group: psychological capital, *p* < 0.01; state self-confidence, *p* < 0.01, older age group < younger age group: motivation to avoid failure, *p* < 0.05
	U16-U18 (older age group)	198	38.8	
Playing position	Guard	179	35.1	Center > guard: reduced sense of accomplishment, *p* < 0.05
	Forward	153	30.0	
	Center	128	25.1	
	Swingman	50	9.8	
Match status	Starter	281	55.1	Starter > substitute: psychological capital, *p* < 0.01; state self-confidence, *p* < 0.01, Starter < substitute: mental fatigue, *p* < 0.01; pre-competition anxiety, *p* < 0.01
	Substitute	229	44.9	
Training years	2–3 years	234	45.9	No significant differences
	4–5 years	186	36.5	
	≥6 years	90	17.6	

### Questionnaire tests

All tests were completed 24 h before the 2024 Sichuan Provincial Adolescent Basketball Championship. Standardized instructions were used, and the tests were administered collectively on-site by trained psychology professionals. To control common method bias, measures such as anonymous filling, reverse scoring of some items, and clear notification that there were no right or wrong answers were adopted, and questionnaires were collected on-site. The following four tests were conducted, respectively.

#### Psychological capital questionnaire

The Psychological Capital Questionnaire (PCQ) developed by Luthans et al. (2007) was adopted. It consists of 20 items across 4 dimensions: self-efficacy (6 items), resilience (5 items), hope (5 items), and optimism (4 items). A 7-point Likert scale was used (1 = strongly disagree, 7 = strongly agree), with a total score ranging from 20 to 140; higher scores indicate higher levels of psychological capital. This questionnaire has been verified by Chinese scholars to be suitable for Chinese athlete populations (Yu et al., 2025). In this study, the Cronbach’s *α* coefficient of the total scale was 0.914, and the *α* coefficients of each dimension ranged from 0.821 to 0.873.

#### Athlete burnout questionnaire

At present, there is a lack of a universally recognized direct mental fatigue scale suitable for Chinese adolescent athletes. The Athlete Burnout Questionnaire (ABQ) (Raedeke and Smith, 2009) has been widely used in sports psychology research to measure mental fatigue-related indicators (emotional exhaustion, reduced sense of accomplishment, devaluation of sport) (Liu et al., 2022), and its reliability and validity have been verified in Chinese athlete groups, so this scale was adopted in this study. It includes 15 items across 3 dimensions: emotional/physical exhaustion (5 items), reduced sense of accomplishment (5 items), and devaluation of sport (5 items). A 5-point scale was adopted (1 = never, 5 = always), with items 1 and 14 scored in reverse. Higher scores on each dimension indicate higher levels of mental fatigue. In this study, the Cronbach’s *α* coefficients of the total scale and each dimension were 0.898, 0.869, 0.841, and 0.792, respectively.

#### Individual differences in achievement tendency

The individual differences in achievement tendency (IDIAT) compiled by Mehrabian (1994) was utilized. It comprises two subscales with a total of 38 items: motivation to approach success (19 items) and motivation to avoid failure (19 items). A 9-point rating scale was used (1 = strongly disagree, 9 = strongly agree), and the total score of achievement motivation was calculated as the score of motivation to approach success minus the score of motivation to avoid failure. This questionnaire has been validated by Chinese scholars for use in Chinese athlete groups (Han et al., 2022). In this study, the Cronbach’s *α* coefficients of the two subscales were 0.854 and 0.862, respectively.

#### Competitive state anxiety inventory-2

The competitive state anxiety inventory-2 (CSAI-2) compiled by Cox et al. (2003) was employed. It contains 27 items across 3 subscales: cognitive state anxiety (9 items), somatic state anxiety (9 items), and state self-confidence (9 items). A 4-point scale was applied (1 = not at all, 4 = very much so). Higher scores on cognitive and somatic anxiety indicate higher anxiety levels, while higher scores on state self-confidence indicate higher levels of confidence. This questionnaire has been confirmed by Chinese scholars to be appropriate for Chinese athlete populations (Zhang et al., 2023). In this study, the Cronbach’s *α* coefficients of the three subscales were 0.921, 0.937, and 0.896, respectively. As a classic calculation method in achievement motivation research (Mehrabian, 1994; Han et al., 2022), the total score of achievement motivation was calculated as the score of motivation to approach success minus the score of motivation to avoid failure, which can effectively reflect individuals’ overall achievement motivation level and motivational orientation.

### Questionnaire validity

Since this study investigated participants’ pre-competition psychological states, a test–retest (randomly selecting 10% of the sample after a 14-day interval) was not conducted to verify questionnaire validity, as it would fail to reflect pre-competition states. To ensure construct validity, confirmatory factor analysis (CFA) was performed for each questionnaire using AMOS 24.0. The fit indices for each scale were acceptable (Supplementary Table S1): *χ*^2^/*df* < 3, IFI/TLI/CFI > 0.90, RMSEA < 0.08. Additionally, average variance extracted (AVE) and composite reliability (CR) were calculated to assess convergent validity. AVE values for all dimensions ranged from 0.51 to 0.68 (≥ 0.50), and CR values ranged from 0.82 to 0.91 (≥ 0.70), confirming good convergent validity (Yu and Cheng, 2024).

To ensure the validity of questionnaire responses, a multi-level guarantee mechanism was established from three aspects: scale selection, test administration procedures, and data screening. Scale selection: Mature scales validated for Chinese athlete groups were adopted; all scales had Cronbach’s *α* coefficients ranging from 0.792 to 0.937, indicating good reliability and strong alignment with the research variables. Test administration: Tests were administered collectively 24 h before competitions by trained psychology professionals using standardized guidelines. Common method bias and social desirability bias were controlled through anonymous filling, reverse scoring of some items, and clarification that there were no right or wrong answers, with the effectiveness of bias control verified via Harman’s single-factor test. Data screening: Strict screening criteria were set to exclude invalid questionnaires with ≥ 3 missing items or 10 consecutive identical responses. The final effective recovery rate reached 98.08%. Combined with logical verification of inter-group differences in demographic variables (e.g., starters had significantly lower anxiety scores than substitutes, and females had higher somatic anxiety than males), the authenticity of questionnaire responses and data validity were further ensured.

### Statistical analysis

SPSS 26.0 was used for data processing to calculate the mean and standard deviation of each variable, and Harman’s single-factor test was performed to control common method bias. Independent-samples *t*-tests and one-way analysis of variance (ANOVA) were used to examine differences in demographic variables. Pearson correlation analysis was conducted to explore relationships between variables. Multiple linear regression analysis was applied to establish prediction models. AMOS 24.0 software was used to construct structural equation models, with sex, age group (U13-U15/U16-U18), and match status (starter/substitute) included as control variables. The Bootstrap method (5,000 resamples) was employed to test mediating effects (Yu and Cheng, 2024). The acceptable criteria for model fit indices were: *χ*^2^/*df* < 3, IFI, TLI, CFI > 0.90, and RMSEA < 0.08. The significance level was set at *α* = 0.05.

## Results

This study is a cross-sectional study with data collected at a single time point (24 h before the competition), so all research results only reflect the correlative relationship between variables, and no causal relationship can be inferred. Descriptive statistics of each variable (Table 2) showed that the total score of athletes’ psychological capital was 73.18 ± 12.45 (moderate to high), with all dimensions of self-efficacy, resilience, hope, and optimism at moderate levels; cognitive state anxiety (22.34 ± 5.87) and somatic state anxiety (21.89 ± 6.23) were at moderate levels, while state self-confidence (24.76 ± 5.42) was slightly higher; the total score of achievement motivation was 8.36 ± 11.24, with motivation to pursue success higher than motivation to avoid failure; the weighted total score of mental fatigue was 0.82 ± 0.31 (mild), and all three dimensions showed moderate performance.

**Table 2:** Descriptive statistics of each variable (*n* = 510).

Variable	Minimum	Maximum	Mean	Standard deviation	Median
Total psychological capital score	35	112	73.18	12.45	74
Self-efficacy dimension	8	36	20.42	4.87	21
Resilience dimension	5	35	17.68	4.32	18
Hope dimension	6	35	19.15	4.56	19
Optimism dimension	4	28	15.93	3.85	16
Motivation to pursue success	48	105	76.52	10.38	78
Motivation to avoid failure	42	98	68.16	10.15	69
Total achievement motivation score	–25	42	8.36	11.24	9
Cognitive state anxiety	10	36	22.34	5.87	22
Somatic state anxiety	9	36	21.89	6.23	22
State self-confidence	12	36	24.76	5.42	25
Total pre-competition anxiety score	21	72	44.23	11.34	44
Emotional/physical exhaustion	6	25	14.87	4.56	15
Reduced sense of accomplishment	5	25	15.32	4.78	15
Devaluation of sport	5	25	15.64	4.92	16
Weighted total mental fatigue score	0.21	1.64	0.82	0.31	0.79

Weighted total mental fatigue score = (emotional/physical exhaustion score + reduced sense of accomplishment score + devaluation of sport score)/(3 × 5), i.e., the average value of each dimension score converted to the 0–1 range. Higher scores indicate higher levels of mental fatigue.

Inter-group differences in demographic variables were as follows (Tables 3–6): female athletes had significantly higher somatic state anxiety than males (*p* < 0.05); athletes in the older age group had significantly higher psychological capital and state self-confidence, and significantly lower motivation to avoid failure than those in the younger age group (*p* < 0.05); centers had significantly higher scores on reduced sense of accomplishment than those of guards (*p* < 0.05); starting athletes had significantly higher psychological capital and state self-confidence, and significantly lower mental fatigue and pre-competition anxiety than substitutes (*p* < 0.01); there were no significant differences among groups with different training years (*p* > 0.05).

**Table 3:** Comparison of variable scores between male and female athletes.

Variable	Male (*n* = 435)	Female (*n* = 75)	*t*	*p*
Total psychological capital score	73.42 ± 12.31	71.78 ± 13.52	1.124	0.262
Motivation to pursue success	76.68 ± 10.42	75.24 ± 10.11	1.087	0.278
Motivation to avoid failure	68.23 ± 10.08	67.52 ± 11.34	0.624	0.533
Total achievement motivation score	8.45 ± 11.18	7.72 ± 11.96	0.532	0.595
Cognitive state anxiety	22.18 ± 5.92	23.24 ± 5.63	−1.456	0.146
Somatic state anxiety	21.65 ± 6.18	23.28 ± 6.01	−2.467	0.014
State self-confidence	24.89 ± 5.38	23.96 ± 5.71	1.423	0.156
Emotional/physical exhaustion	14.76 ± 4.61	15.38 ± 4.42	−1.187	0.236
Reduced sense of accomplishment	15.24 ± 4.82	15.96 ± 4.65	−1.302	0.194
Devaluation of sport	15.58 ± 4.88	16.12 ± 5.06	−0.894	0.372

**Table 4:** Comparison of psychological capital, state self-confidence, and motivation to avoid failure scores across age groups.

Variable	Younger age group (*n* = 312)	Older age group (*n* = 198)	*F*	*p*
Total psychological capital score	71.85 ± 12.63	75.24 ± 11.78	5.628	0.004
State self-confidence	23.68 ± 5.56	26.48 ± 4.92	8.132	<0.001
Motivation to avoid failure	69.34 ± 10.24	66.47 ± 9.87	3.412	0.034

**Table 5:** Comparison of reduced sense of accomplishment scores across playing positions.

Position	*n*	Reduced sense of accomplishment	*F*	*p*
Guard	179	14.98 ± 4.82	3.015	0.030
Forward	153	15.18 ± 4.71		
Center	128	16.42 ± 4.68		
Swingman	50	15.36 ± 4.95		

**Table 6:** Comparison of key variable scores between starters and substitutes.

Variable	Starter (*n* = 281)	Substitute (*n* = 229)	*t*	*p*
Total psychological capital score	74.96 ± 12.08	70.82 ± 12.81	3.127	0.002
State self-confidence	25.68 ± 5.23	23.64 ± 5.48	4.256	<0.001
Total mental fatigue score	0.76 ± 0.28	0.89 ± 0.33	−2.843	0.005
Total pre-competition anxiety score	42.15 ± 10.68	46.87 ± 11.89	−2.634	0.001

Correlation analysis (Table 7) indicated that psychological capital was positively correlated with state self-confidence (*r* = 0.482), and negatively correlated with state anxiety and mental fatigue (*r* = −0.498 ~ −0.286); mental fatigue was positively correlated with state anxiety (*r* = 0.376 ~ 0.389); achievement motivation was positively correlated with state self-confidence (*r* = 0.315) and negatively correlated with state anxiety (*r* = −0.178 ~ −0.192), which met the conditions for mediation testing.

**Table 7:** Pearson correlation matrix of key variables (*n* = 510).

Variable	1	2	3	4	5	6
1. Psychological capital	1					
2. Mental fatigue	−0.286**	1				
3. Achievement motivation	0.228**	−0.156*	1			
4. State self-confidence	0.482**	−0.452**	0.315**	1		
5. Cognitive state anxiety	−0.498**	0.376**	−0.192**	−0.628**	1	
6. Somatic state anxiety	−0.512**	0.389**	−0.178*	−0.645**	0.734**	1

*p < 0.05, **p < 0.01.

**Figure 1:**
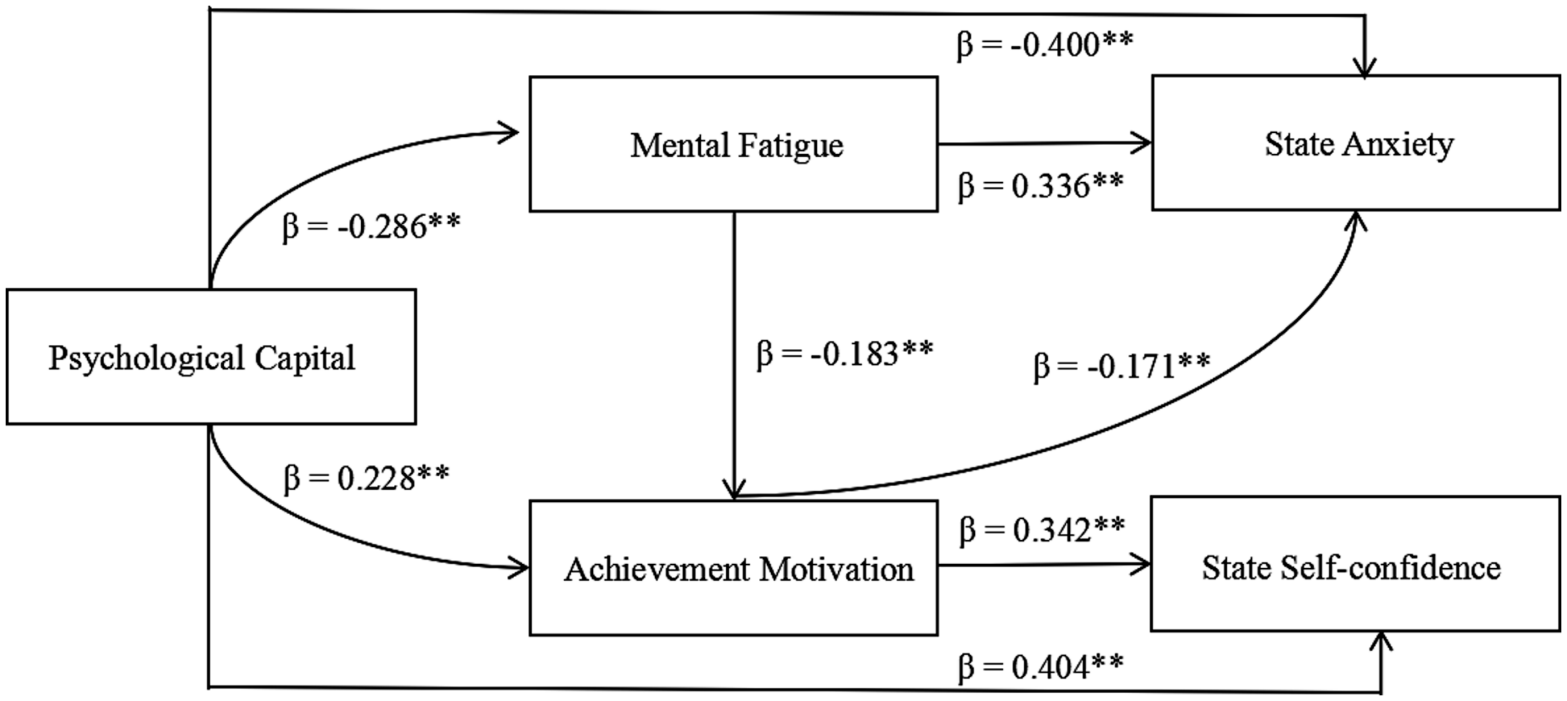
Chain mediation model diagram (standardized path coefficients) Figure displays the chain mediation model with standardized path coefficients (all significant paths *p* < 0.01), and the model fit indices are excellent (*χ*^2^/*df* = 2.134, IFI = 0.992, TLI = 0.985, CFI = 0.992, RMSEA = 0.047), clearly reflecting the direct path, independent mediating paths, and chain mediating path of all variables.

Mediation effect tests (Table 8, Figure 1) revealed that: (1) The direct effect of psychological capital on state anxiety was significant (direct effect = −0.400, 95% CI [−0.482, −0.318], accounting for 73.29%), and its direct effect on state self-confidence was also significant (direct effect = 0.404, 95% CI [0.323, 0.485], accounting for 83.82%); (2) The independent mediating effect of mental fatigue (psychological capital → mental fatigue → state anxiety) was significant (indirect effect = −0.096, 95% CI [−0.148, −0.052], accounting for 17.55%); (3) The independent mediating effects of achievement motivation were significant both between psychological capital and state anxiety (psychological capital → achievement motivation → state anxiety; indirect effect = −0.039, 95% CI [−0.072, −0.011], accounting for 7.13%) and between psychological capital and state self-confidence (psychological capital → achievement motivation → state self-confidence; indirect effect = 0.078, 95% CI [0.041, 0.122], accounting for 16.18%); (4) the chain mediating effect of mental fatigue and achievement motivation (psychological capital → mental fatigue → achievement motivation → state anxiety) was statistically significant (indirect effect = 0.011, 95% CI [0.003, 0.024], accounting for 2.01%), but the effect size was small. This suggests that while the sequential path is theoretically meaningful, its practical impact on pre-competition anxiety is limited compared to the direct effect of psychological capital (73.29%) and the independent mediating effect of mental fatigue (17.55%). (5) The total effect of psychological capital on state anxiety was significant (total effect = −0.547, 95% CI [−0.621, −0.473]), and its total effect on state self-confidence was also significant (total effect = 0.482, 95% CI [0.405, 0.559]) (see Table 9).

**Table 8:** Path coefficients and effect decomposition of the mediation model with mental fatigue or achievement motivation.

Effect type	Specific path/relationship	Standardized coefficient	Effect value	95% CI	Effect proportion	*p*
Direct effect	Psychological capital → state anxiety	−0.400	−0.400	[−0.482, −0.318]	73.29%	<0.001
Direct effect	Psychological capital → state self-confidence	0.404	0.404	[0.323, 0.485]	83.82%	<0.001
Independent mediation effect (mental fatigue)	Psychological capital → mental fatigue → state anxiety	−0.096	−0.096	[−0.148, −0.052]	17.55%	<0.001
Independent mediation effect (achievement motivation)	Psychological capital → achievement motivation → state anxiety	−0.039	−0.039	[−0.072, −0.011]	7.13%	<0.001
Independent mediation effect (achievement motivation)	Psychological capital → achievement motivation → state self-confidence	0.078	0.078	[0.041, 0.122]	16.18%	<0.001
Chain mediation effect	Psychological capital → mental fatigue → achievement motivation → state anxiety	0.011	0.011	[0.003, 0.024]	2.01%	0.008
Total effect	Psychological capital → state anxiety	−0.547	−0.547	[−0.621, −0.473]	100.00%	<0.001
Total effect	Psychological capital → state self-confidence	0.482	0.482	[0.405, 0.559]	100.00%	<0.001

**Table 9:** Effect decomposition of the chain mediation model.

Effect path	Indirect effect value	95% CI	Effect proportion
Psychological capital → mental fatigue → achievement motivation → state anxiety	0.011	[0.003, 0.024]	2.01%

## Discussion

This study aimed to explore the internal mechanism by which psychological capital is associated with pre-competition anxiety in adolescent basketball players, with a focus on examining the independent and chain mediating roles of mental fatigue and achievement motivation.

### Correlation and main effect analysis among variables

H1 was verified, meaning there were significant pairwise correlations among the four variables of psychological capital, mental fatigue, achievement motivation, and pre-competition anxiety. First, psychological capital was significantly positively correlated with state self-confidence and significantly negatively correlated with state anxiety, and its direct effects on state self-confidence and state anxiety were both significant, which was consistent with previous studies (Lee et al., 2022; Domínguez et al., 2024), confirming the core role of psychological capital as a positive psychological resource in improving athletes’ self-confidence and alleviating anxiety. Second, the negative correlation between psychological capital and mental fatigue, as well as the positive correlation between mental fatigue and state anxiety, were in line with the basic viewpoint of the Conservation of Resources Theory, i.e., individuals are more prone to exhaustion when lacking resources, and exhaustion directly exacerbates anxiety experiences (Strack et al., 2015). In addition, the positive correlation between achievement motivation and state self-confidence suggested that the motivational tendency to pursue success had a positive promoting effect on pre-competition self-confidence.

### Independent mediating roles of mental fatigue and achievement motivation

H2 was verified, indicating that mental fatigue and achievement motivation played independent mediating roles between psychological capital and pre-competition anxiety, respectively. Psychological capital can indirectly alleviate athletes’ anxiety by reducing mental fatigue levels (Oliver et al., 2025). This path confirmed the Conservation of Resources Theory. Athletes with high levels of psychological capital have traits such as self-efficacy, resilience, and optimism that help them cope with training and competition pressures more effectively, thereby reducing excessive consumption of psychological resources and maintaining a relatively low level of mental fatigue (Proost et al., 2022). A lower level of mental fatigue enables athletes to maintain better emotional regulation ability before competitions and exhibit lower anxiety (Strack et al., 2015). The discovery of this mediating path (accounting for 17.55% of the total effect) suggests that helping athletes accumulate psychological capital is an effective way to prevent mental fatigue and alleviate pre-competition anxiety in psychological interventions. Meanwhile, psychological capital can also indirectly improve state self-confidence and alleviate anxiety by optimizing the structure of achievement motivation (Khajavy et al., 2019). This study holds that athletes with high psychological capital are more inclined to set challenging goals and firmly believe in their ability to succeed, showing stronger motivation to pursue success. This positive motivational orientation can enhance pre-competition self-confidence, and the corresponding mediating effect accounts for 7.13% of the total effect on state anxiety and 16.18% on state self-confidence, indicating that it is an important buffer against anxiety. Therefore, cultivating athletes’ psychological capital is of great significance for guiding them to form healthy achievement goal orientations and promoting positive pre-competition psychological states. The optimization of achievement motivation cannot only improve athletes’ pre-competition self-confidence, but also promote their active participation in training and competition, reduce sedentary behavior and improve sleep quality (El Oirdi et al., 2021; El Oirdi et al., 2023b), thus indirectly alleviating pre-competition anxiety.

### Chain mediating role of mental fatigue and achievement motivation

H3 was verified, meaning mental fatigue and achievement motivation constituted a chain mediating path between psychological capital and pre-competition anxiety, indicating the negative impact of psychological capital on pre-competition anxiety. The sequential order of mental fatigue and achievement motivation in the chain mediation model is grounded in the logical relationship of resource loss preceding motivation change in the Conservation of Resources Theory, and basic psychological needs underpinning motivation formation in Self-Determination Theory. Mental fatigue, as a direct manifestation of psychological resource loss, is a prerequisite for changes in achievement motivation; only when athletes are in a state of low mental fatigue can their basic psychological needs be met, and healthy achievement motivation be subsequently stimulated (Milyavskaya et al., 2021; Proost et al., 2022).

Sufficient psychological capital first exerts a resource protection role, which can effectively reduce the psychological consumption caused by high-intensity training (Chen et al., 2024; Xu et al., 2017), enabling athletes to maintain a low level of mental fatigue. When athletes are away from an exhausted state, their basic psychological needs such as intrinsic interest in basketball, sense of competence, and autonomy are more likely to be satisfied, which creates necessary conditions for stimulating and maintaining healthy achievement motivation oriented towards pursuing success. This study argues that the positive and autonomous motivational state driven by the satisfaction of intrinsic needs has become a solid psychological foundation for athletes to remain calm and confident in high-pressure competition environments. For substitute players or younger athletes with high levels of mental fatigue, if psychological interventions only emphasize building self-confidence or stimulating the desire for success while ignoring first supplementing their psychological resources and alleviating their mental exhaustion, the intervention effect may be poor. Therefore, priority should be given to helping athletes cope with pressure and restore psychological energy, and then guiding them to establish healthy achievement goals. Given that the Athlete Burnout Questionnaire primarily assesses chronic psychological exhaustion rather than acute pre-competitive mental fatigue, the observed sequential mediation effect of mental fatigue should be interpreted with this conceptual distinction in mind.

It is worth noting that the demographic differences in this study revealed that female athletes had significantly higher somatic state anxiety than males, which may be related to their higher sensitivity to physical sensations and weaker ability to regulate pre-competition physiological arousal (Sánchez et al., 2023). This suggests the need to strengthen pre-competition physical relaxation training for female athletes. Additionally, centers had significantly higher scores on reduced sense of accomplishment than guards, which may stem from their on-court role being more dependent on team cooperation and having relatively fewer scoring opportunities, leading to a decline in self-efficacy. It is necessary to clarify the core value of centers in defense, rebounding, and coordination, and strengthen positive feedback on “non-scoring success experiences”; the psychological differences between starting and substitute athletes (starters have higher psychological capital and lower anxiety) may be related to starters accumulating more successful experiences, while substitute athletes need more actual combat opportunities to accumulate confidence through the achievement of phased small goals.

This study has limitations. First, this study adopts a cross-sectional research design with data collected only 24 h before the competition, which cannot reveal the dynamic change process of variables (Cheng et al., 2023) and infer the causal relationship between psychological capital, mental fatigue, achievement motivation and pre-competition anxiety. Future studies can adopt a longitudinal tracking design or experimental intervention design to collect multi-time point data and verify the causal relationship between variables. Second, this study has a gender imbalance in the sample, with only 14.7% female adolescent basketball players included. The small sample size of female athletes may lead to the inability to fully reveal the psychological characteristics of female basketball players, and the research conclusions on female athletes need to be further verified by expanding the female sample size in future studies. Finally, this study uses the Athlete Burnout Questionnaire to measure mental fatigue, and there is a certain deviation between the measurement tool and the research variable (chronic burnout vs. acute mental fatigue). Future studies can select or develop a special direct mental fatigue scale for adolescent athletes to improve the structural validity of the measurement.

## Conclusion

Based on the correlative relationship revealed by cross-sectional data, this study confirms that psychological capital of adolescent basketball players is directly and negatively associated with pre-competition anxiety, and also indirectly alleviates pre-competition anxiety by reducing mental fatigue and optimizing achievement motivation, with mental fatigue and achievement motivation playing both independent and chain mediating roles. Based on the demographic differences in the study, targeted interventions are proposed for different subgroups: improve psychological capital and reduce failure-avoidance motivation for younger athletes; alleviate reduced sense of accomplishment for centers; strengthen pre-competition physical relaxation training for females; and provide more competitive opportunities for substitutes to build self-confidence.

## Data Availability

The raw data supporting the conclusions of this article will be made available by the authors, without undue reservation.
