# Supplementary material for: Psychological capital and pre-competition anxiety among adolescent basketball players: the chain mediating roles of mental fatigue and achievement motivation
Source: Front Psychol. 2026 Mar 6;17:1800210. doi: 10.3389/fpsyg.2026.1800210 (PMC13003534; doi:10.3389/fpsyg.2026.1800210)
Supplement: Supplementary file 1 [file Table_1.doc]

Table S1 Confirmatory Factor Analysis Fit Indices of All Scales

| Scale Name | χ²/df | IFI | TLI | CFI | RMSEA |
| --- | --- | --- | --- | --- | --- |
| Psychological Capital Questionnaire (PCQ) | 2.35 | 0.94 | 0.93 | 0.94 | 0.052 |
| Athlete Burnout Questionnaire (ABQ) | 2.18 | 0.95 | 0.94 | 0.95 | 0.048 |
| Individual Differences in Achievement Tendency (IDIAT) | 2.62 | 0.92 | 0.91 | 0.93 | 0.058 |
| Competitive State Anxiety Inventory-2 (CSAI-2) | 2.41 | 0.94 | 0.93 | 0.94 | 0.054 |

Note: All fit indices meet the acceptable criteria for structural equation modeling (χ²/df < 3, IFI/TLI/CFI > 0.90, RMSEA < 0.08)
